# Role of Milk Intake in Modulating Serum Lipid Profiles and Gut Metabolites

**DOI:** 10.3390/metabo14120688

**Published:** 2024-12-07

**Authors:** Ting Xu, Chang Zhang, Yufeng Yang, Liang Huang, Qingyou Liu, Ling Li, Qingkun Zeng, Zhipeng Li

**Affiliations:** 1 Guangxi Zhuang Autonomous Region Buffalo Milk Quality and Safety Control Technology Engineering Research Center, Guangxi Buffalo Research Institute, Chinese Academy of Agricultural Sciences, Nanning 530001, China; xut_09@163.com (T.X.); lling2010@163.com (L.L.); 2 Guangxi Key Laboratory of Animal Reproduction, Breeding and Disease Control, College of Animal Science and Technology, Guangxi University, Nanning 530004, China; zc_6537@163.com (C.Z.); yangyufeng@st.gxu.edu.cn (Y.Y.); 3 School of Animal Sciences, Zhejiang University, Hangzhou 310058, China; 12317046@zju.edu.cn; 4 Guangdong Provincial Key Laboratory of Animal Molecular Design and Precise Breeding, School of Life Science and Engineering, Foshan University, Foshan 528225, China; qyliu-gene@fosu.edu.cn

**Keywords:** milk, metabonomic, blood lipid, buffalo

## Abstract

Background/Objectives: Milk is one of the main sources of nutrition in people’s daily diet, but the fat in milk raises health concerns in consumers. Here, we aimed to elucidate the impact of Buffalo milk and Holstein cow milk consumption on blood lipid health through metabolomics analysis. Methods: Golden hamsters were administered Murrah Buffalo milk (BM) or Holstein cow milk (HM), and the body weight and serum lipid indicators were tested and recorded. The hamsters receiving equal amounts of physiological saline were used as the negative control (NC). Serum and fecal samples were collected, and LC-MS was used to identify the metabolites in the samples. Results: The results showed that both the BM and HM groups exhibited a significant reduction in body weight compared to that of the NC group from day 9, and the serum TG, TC, and LDL-C levels were significantly lower than those of the NC group. Further analysis identified 564 and 567 metabolites in the serum and fecal samples shared in the BM and HM groups and significantly different from those in the NC group, which were mainly enriched in the pathways related to lipid metabolism, such as fatty acid biosynthesis, arachidonic acid metabolism, and primary bile acid biosynthesis. Correlation analysis further suggested that milk intake can increase the levels of Muramic Acid, Oleoyl Ethanolamide, Seratrodast, Chenodeoxycholic Acid, Docosahexaenoic Acid Ethyl Ester, and Deoxycholic Acid in the serum and gut microbiota, which may affect TG, TC, HDL-C, and LDL-C in the serum, and thereby benefit the body’s lipid health. Conclusions: The results further confirmed that milk intake has a beneficial effect on blood lipid health by altering multiple metabolites in the serum and the gut. This study provides novel evidence that milk consumption is beneficial to health and is a reference for guiding people to a healthy diet.

## 1. Introduction

Milk is one of the main sources of nutrition in people’s daily diet because it is rich in fat, proteins, lactose, vitamins, and minerals [1] and is a recommended food listed in the Dietary Guidelines of many countries. It is considered as an invaluable and irreplaceable food that offers long-term benefits to people of all ages [2]. However, there are some controversies regarding the consumption of milk, especially due to the fat in milk, among adults [3]. It is well known that the excessive intake of fat in the diet is one of the major factors leading to dyslipidemia. And elevated levels of triglycerides (TGs), total cholesterol (TC), and low-density lipoprotein cholesterol (LDL-C) in serum are the key factors leading to blood lipid abnormalities, increasing the risk of metabolic diseases, such as obesity, hyperlipidemia, and hypertension [4,5]. However, epidemiologic studies reinforce the possible role of milk consumption in preventing cardiovascular diseases (CVDs), some forms of cancer, obesity, and diabetes [6]. Therefore, revealing the effects of milk intake on hosts’ energy and lipid metabolism may provide an essential reference for guiding people to a healthy diet.

The complex macromolecules in food are usually digested and hydrolyzed into absorbable small molecules, and then absorbed into the blood, affecting the metabolism and health of the host. Dietary components are a fundamental source of the plasma metabolome, and individual dietary habits can predict the levels of specific metabolites in plasma [7]. In addition, the intake of different kinds of foods can alter the gut flora, and then indirectly affect the host’s health through microbial metabolism. The gut has been found to play crucial roles in maintaining physiological and metabolic homeostasis through microbial-derived metabolites. For example, a previous study assessed 1183 plasma metabolites in 1368 extensively phenotyped individuals from the Lifelines DEEP and Genome of the Netherlands cohorts and found the proportion of inter-individual variation in the plasma metabolome explained by different factors, characterizing 610, 85, and 38 metabolites as dominantly associated with diet, the gut microbiome, and genetics, respectively [7]. Feeding diet-induced obese mice two servings of yogurt daily can prevent the development of insulin resistance and hepatic steatosis [8]. And based on a mouse model, the beneficial effects of yogurt on insulin sensitivity are driven by changes in the composition and function of the gut microbiota, as demonstrated by fecal transplantation studies [8]. The high-level consumption of fermented dairy products has been found to increase the *Akkermansia* and major short-chain fatty acid contents in the gut and reduce the serum malondialdehyde levels [9]. And a high-cholesterol diet has been confirmed to induce dysbiosis and alterations in gut bacterial metabolites, which thereby harm a person’s health [10]. Therefore, a healthy diet is essential to keep healthy, but how diet affects health is still not fully understood.

Recently, metabolomics has been increasingly applied to the study of metabolic diseases. Buffalo milk contains higher levels of milk fat, proteins, total solids, and solid non-fat than Holstein cow milk [11], and the higher milk fat and protein contents make it widely welcomed by consumers. However, few studies have focused on the effects of Buffalo milk intake on body lipid metabolism. Golden hamsters have a similar lipid metabolism pattern to that of humans, which has been widely used in the modeling of human hyperlipidemia [12]. They can better present the enzyme pathway of fat synthesis and metabolism and can be used to reliably predict the effects of hyperlipidemia on humans’ serum cholesterol clearance capacity and lipid profile regulation [13,14]. Here, golden hamsters were supplemented with Holstein or Buffalo milk, and the metabolites in the sera and the gut microbes were systemically analyzed. This study may provide a novel understanding of the effect of milk consumption on health and a reference for guiding people to a healthy diet.

## 2. Materials and Methods

### 2.1. Animal Experiment Design

Male LVG hamsters (~130 g; 7 weeks) were purchased from Beijing Vitalstar Biotechnology Co., Ltd. under permission number SYXK (Beijing, China) 2022-0052 and housed under standard laboratory conditions (20–22 °C; light/dark cycle for 12 h:12 h). All the animals were treated humanely as outlined in the Guide for the Care and Use of Experimental Animals of the National Institutes of Health, and the protocol was approved by the Animal Experiment Ethics Committee of Guangxi University (GXU-2023-0034). The hamsters were randomly divided into three groups (N = 10) and kept a normal diet. Meanwhile, one group was given normal saline (defined as NC group), one group was given Murrah Buffalo milk (defined as BM group), and the other group was given Holstein cow milk (defined as HM group). The normal diet in this study was purchased from Trophic Animal Feed High-tech Co., Ltd. (Nantong, China). The normal diet comprised 5% fat and 15 % protein, and the energy value was 4200 kcal/kg (Table S1). In this study, except for a specific test, all the animals were freely allowed food and water. Buffalo milk and Holstein cow milk was collected from local farms (Royal Group Co., Ltd.) in Nanning, China. All the milk samples were collected in triplicate and the routine composition (lactose, fat, protein, total solid, and non-fat solid) was analyzed using a multifunction analyzer for dairy products (MilkoScan FT-120, FOSS Electric A/S, Hillerod, Denmark). Based on the Chinese Dietary Guidelines (2022), adults are recommended to consume 300–500 mL of milk or dairy products per day [15]. The dose of Buffalo milk and Holstein cow milk for hamsters was calculated based on a previous report [16], and the dose was set as 2 mL/day (1 mL in the morning and 1 mL in the night). All milk was pasteurized before use. Milk was given as a separate food item. Milk was injected directly into the hamster’s stomach by gavage to ensure that all milk was consumed. The hamsters’ feed intake was recorded daily. Body weight was measured every three days. Blood samples were collected from the orbital vein every two weeks, and serum was separated for further analysis. Stool samples were collected at the end of the experiment and stored at −80 °C until further study. All the hamsters were euthanatized by carbon dioxide inhalation after 8-week experiments.

### 2.2. Biochemical Assays

Fasting blood samples of 30 hamsters (1 mL blood per hamster) were collected every 2 weeks. A total of 2 mL of blood was collected before sacrificing the animals and was used for biochemical tests and serum metabolome sequencing. The hamsters were anaesthetized with diethyl ether, and blood was collected retro-orbitally in fresh Eppendorf tubes. The blood samples were kept at room temperature for 45 min, and then centrifuged at 3000 rpm at 4 °C for 15 min to separate the serum samples. The serum samples were stored at −80 °C until analysis. The levels of TG, TC, HDL-C, and LDL-C in the serum were measured using an automated animal biochemical analyzer (URIT, 8021AVet, Guilin, China).

### 2.3. LC-MS Analysis

Fecal water was extracted by taking a weighed sample of thawed stool and mixing with methanol in a ratio of 3 mL/g. The mixture was homogenized using vortexing for 60 s, and then centrifuged at 10,000 rpm for 10 min. Supernatants were transferred to clean the tubes, and then filtered through a membrane (0.2 μm pore size) [17,18]. Finally, the supernatants were collected and loaded onto the LC-MS system for analysis. The chromatographic separation conditions used in this study referred to some previous reports [19,20]. In brief, equal volumes from each sample were mixed as quality control (QC) samples. UHPLC–MS spectrometry was performed using a Vanquish UHPLC system (Thermo Fisher, Germering, Germany) combined with an Orbitrap Q ExactiveTM HF-X mass spectrometer (Thermo Fisher, Germering, Germany). The samples were loaded onto a Hypersil Gold column (100 × 2.1 mm, 1.9 μm) at a flow rate of 0.2 mL/min, with a linear gradient of 17 min. The positive polarity eluents were eluent A (0.1% formic acid (FA) in water) and eluent B (methanol). The negative polarity eluents were eluent A (5 mM ammonium acetate, pH 9.0) and eluent B (methanol). The solvent gradient was set as follows: 2% B, 1.5 min; 2–100% B, 12.0 min; 100% B, 14.0 min; 100–2% B, 14.1 min; and 2% B, 17 min [19,20]. The serum samples were directly loaded onto the LC-MS system and analyzed as described above.

### 2.4. Non-Targeted Metabolomic Analysis

Raw data files generated by UHPLC-MS/MS were processed using Compound Discoverer 3.1 (CD3.1, Thermo Fisher) with the following non-targeted metabolomic workflow: spectra selection, blank subtraction, peak picking and retention time (RT) alignment, and candidate comparison with the mzCloud and ChemSpider databases. The processing and analysis of mass spectrometry data used Compound Discoverer 3.0 (CD 3.0) software. The data were analyzed using the SIMCA-P 14.1 software package (Umetrics, Umeå, Sweden), including principal component analysis (PCA) and orthogonal partial least-squares discriminant analysis (OPLS-DA). Variable importance in projection (VIP) was obtained from the OPLS-DA model, defining VIP value > 1 and *p* < 0.05 as differential metabolites. MetaboAnalyst 5.0 was used for KEGG pathway analysis, and a value of *p* < 0.05 was used to indicate statistical significance.

### 2.5. Statistical Analysis

Statistical analysis was performed using SPSS 29.0 software (Chicago, IL, USA). Normal distribution within the sample groups was determined using the D’Agostino and Pearson normality test. Two-way ANOVA was used when comparing more than two groups and/or more than two independent variables, such as time and treatment. The data are presented as the means ± standard error (SEM). *p* < 0.05 was considered to be a statistically significant difference. Different letters indicate significant differences (*p* < 0.05), while the same letters indicate insignificant differences (*p* > 0.05).

## 3. Results

### 3.1. The Impact of Buffalo Milk and Holstein Cow Milk Intake on Blood Lipid in Golden Hamsters

**Figure 1:**
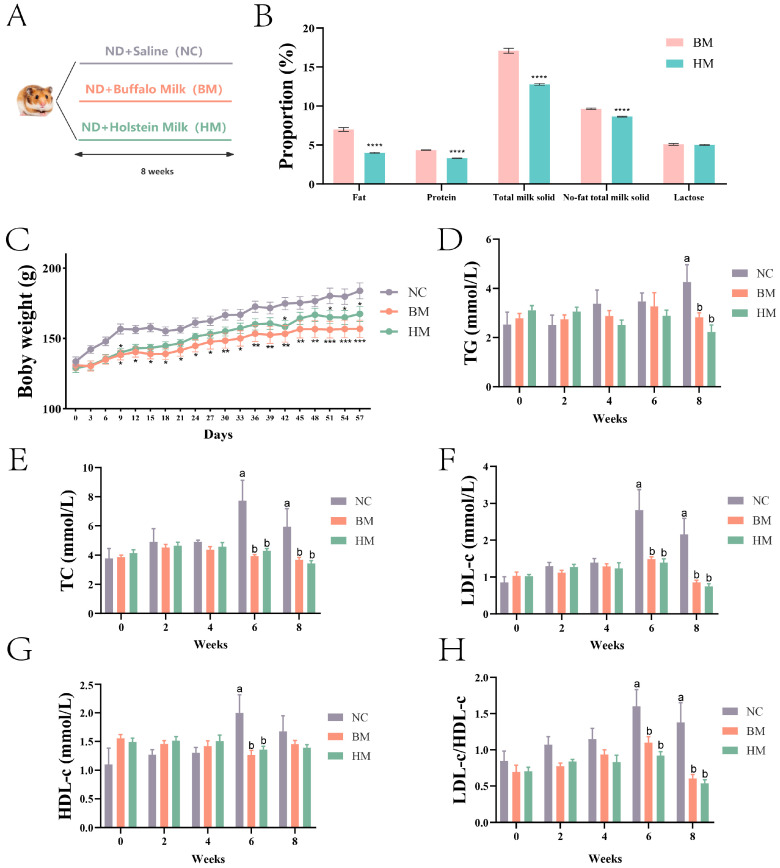
The effects of Buffalo milk and Holstein cow milk intake on the body weight and blood lipid levels of the golden hamsters. (**A**) The animal experimental design. (**B**) The analysis of the composition of Buffalo milk and Holstein cow milk. (**C**) The body weight of the hamsters. (**D**) The serum TG levels of the hamsters. (**E**) The serum TC levels of the hamsters. (**F**) The serum LDL-c levels of the hamsters. (**G**) The serum HDL-c levels of the hamsters. (**H**) The serum LDL-c/HDL-c ratio of the hamsters. Different labels (*, **, ***, ****) indicate significant differences (*p* < 0.05, *p* < 0.01, *p* < 0.001, *p* < 0.0001), respectively. The different labels (a and b) indicate significant differences (*p* < 0.05).

The hamsters were divided into three groups and given Buffalo milk or Holstein cow milk for 8 weeks (Figure 1A). The analysis of milk composition revealed that Holstein cow milk contains significantly lower levels of fat, protein, total solids, and non-fat solids compared to those in Buffalo milk, while the fat content in Buffalo milk is twice as high as that in Holstein cow milk. No significant difference was found in the lactose content (Figure 1B, Table S2). Body weight was measured every three days, and results showed that both the BM and HM groups exhibited a significant reduction in body weight compared to that of the NC group from day 9 (Figure 1C, Table S3). Further analysis showed that the serum TG content in the NC group was significantly higher than those in the HM and BM groups on week 8 (Figure 1D, Table S4). The serum TC and LDL-C contents in the NC group were significantly higher than those in the HM and BM group from week 6 (Figure 1E,F, Table S4). The serum HDL-C content in the NC group was significantly higher than those in the HM and BM group on week 6, but decreased to a comparable level with those of the HM and BM group on week 8 (Figure 1G, Table S4). The LDL-c/HDL-c ratio in the NC group was also significantly higher than that in the BM and HM groups from 6 week (Figure 1H, Table S4), which further suggest the beneficial effect of milk consumption. No significant difference in the TG, TC, HDL-C, and LDL-C contents and the LDL-c/HDL-c ratio were found between the HM and BM groups. The results suggest that intake of milk can reduce body weight and the serum TG, TC, and LDL-c contents, which is beneficial for blood lipid health.

### 3.2. Effect of Buffalo Milk and Holstein Cow Milk Intake on Serum Metabolites

**Figure 2:**
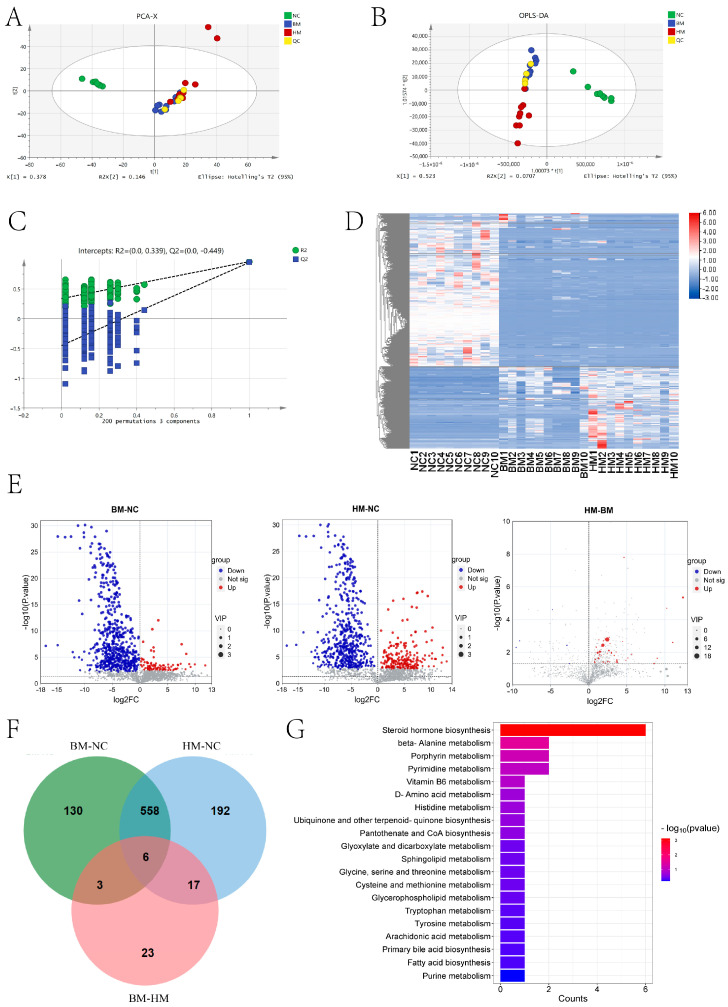
The effects of Buffalo milk and Holstein cow milk intake on the serum metabolites in the golden hamsters. (**A**) The principal component analysis (PCA) of the metabolites. (**B**) The OPLS-DA analysis of the metabolites. (**C**) The OPLS-DA permutation test of the metabolomics. (**D**) A heatmap of the cluster analysis of the metabolomics. (**E**) The identification of differential metabolites. (**F**) A Venn diagram showing the differential metabolites between the BM vs. NC, HM vs. NC, and HM vs. BM groups. (**G**) The KEGG pathway enrichment analysis of the differential metabolites shared in both the BM and HM groups and significantly different from those in the NC group.

We identified 1684 metabolites in the serum (Table S5). Principal component analysis (PCA) was conducted based on the metabolomic data, revealing that PC1 and PC2 explained 37.8% and 14.6% of the variance, respectively (Figure 2A). Further orthogonal partial least squares discriminant analysis (OPLS-DA) indicated significant separation among the three groups of metabolites (Figure 2B). We performed permutation test 200 times, and the results showed that the original R2 and Q2 values were consistently higher than the permuted R2 and Q2 values (Figure 2C), indicating the reliability of the analysis model. Clustering analysis demonstrated the distinct clustering of differential metabolites between the NC group and the BM and HM groups (Figure 2D). Among the metabolites, 697 significantly different metabolites (DEMs) were identified between the BM and NC groups, including 92 significantly upregulated metabolites and 605 significantly downregulated metabolites in the BM group (Figure 2E,F). Between the HM and NC groups, nearly 46% (773/1685) of the metabolites were found to be significantly different, including 223 significantly upregulated metabolites and 550 significantly downregulated metabolites in the HM group (Figure 2E,F). Between the HM and BM groups, 49 DEMs were identified, including 44 significantly upregulated metabolites and 5 significantly downregulated metabolites in the HM group (Figure 2E,F). Combining the metabolites data of the serum, 564 metabolites were found to be shared both in the BM and HM groups and significantly different from those in the NC group (Figure 2F). Further KEGG analysis revealed that these DEMs were enriched in 20 KEGG pathways, including the pathways related to lipid metabolism, such as fatty acid biosynthesis, arachidonic acid metabolism, and primary bile acid biosynthesis (Figure 2G). The results showed that milk intake significantly altered the serum metabolites in the hamsters, especially affecting the pathways for primary bile acid biosynthesis, niacin, and fatty acid biosynthesis, and arachidonic acid metabolism.

### 3.3. Effect of Buffalo Milk and Holstein Cow Milk Intake on Gut Microbial Metabolites

**Figure 3:**
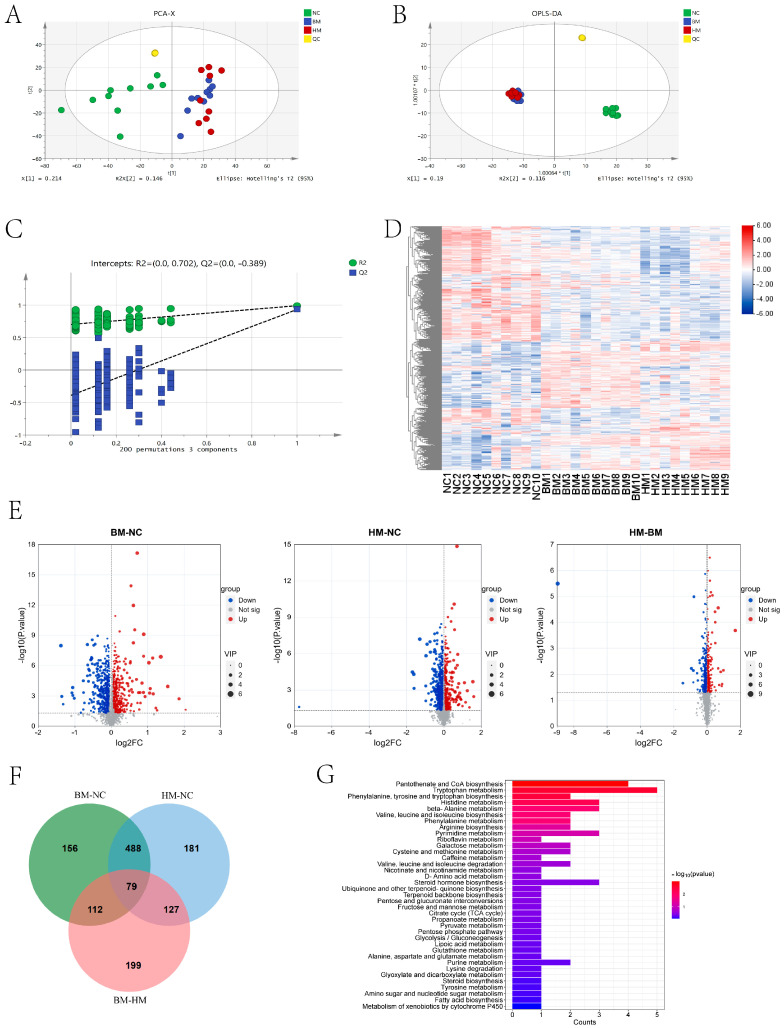
The effects of Buffalo milk and Holstein cow milk intake on the gut microbial metabolites in the golden hamsters. (**A**) The principal component analysis (PCA) of the NC, BM, and HM groups. (**B**) An OPLS-DA plot comparing the three groups. (**C**) The OPLS-DA permutation test for gut microbiota metabolomics. (**D**) A heatmap of the cluster analysis among the three groups. (**E**) The visualization of differential metabolites. (**F**) A Venn diagram showing the union of differential metabolites between the BM vs. NC, HM vs. NC, and HM vs. BM groups. (**G**) The KEGG pathway enrichment analysis of the differential metabolites shared both in the BM and HM groups and significantly different from those in the NC group.

Using LC/MS, we identified 3130 metabolites in the gut microbiome (Table S6). Principle component analysis (PCA) was performed based on the metabolites profiling data, and the results showed that PC1 and PC2 explained 21.4% and 14.6% of the variation, respectively (Figure 3A). Further OPLS-DA showed that the metabolites of the BM and HM groups were significantly separated from those in the NC group (Figure 3B). We performed permutation test analyses 200 times and found that the original R2 and Q2 are always greater than those after permutation (Figure 3C), suggesting that this analytic model is reliable for further analysis. Cluster analysis indicated the significant clustering of differential metabolites between the NC group and the BM and HM groups (Figure 3D). Among the metabolites, 835 differential metabolites (DEMs) were identified between the BM and NC group, including 365 significantly upregulated metabolites and 470 significantly downregulated metabolites in the BM group (Figure 3E,F). Eight hundred and seventy-five DEMs were identified between the HM and NC group, including three hundred and fifteen significantly upregulated metabolites and five hundred and sixty significantly downregulated metabolites in the HM group (Figure 3E,F). Five hundred and seventeen DEMs were identified between the HM and BM groups, including two hundred and seventeen significantly upregulated metabolites and three hundred significantly downregulated metabolites in the HM group (Figure 3E,F). Combining the metabolites data of the serum, 567 metabolites were found to be shared both in the BM and HM groups and significantly different from those in the NC group (Figure 3F). Further KEGG analysis showed that these DEMs were enriched in 37 pathways, such as those for nicotinate and nicotinamide metabolism, tryptophan metabolism, and fatty acid biosynthesis related to lipid metabolism (Figure 3G). The results suggested that milk intake can alter the composition of gut microbial metabolites, with differential metabolites enriched in the pathways related to blood lipid health, including nicotinate and nicotinamide metabolism, tryptophan metabolism, and fatty acid biosynthesis.

### 3.4. Correlation Analysis of Metabolomics and Blood Lipid

**Figure 4:**
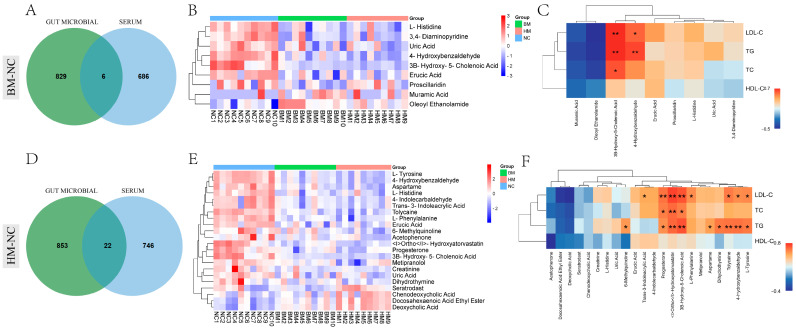
Correlation analysis of shared differential metabolites from serum and gut microbiota with blood lipids between BM and HM groups compared to those of NC group. (**A**) Venn diagram of shared gut microbial and serum differentia metabolites in BM group compared to NC group. (**B**) Cluster analysis of shared DEMs in NC, BM, and HM groups. (**C**) Pearson correlation analysis between shared differential metabolites and serum lipid parameters. (**D**) Venn diagram of shared gut microbial and serum differential metabolites in HM group compared to NC group. (**E**) Cluster analysis of shared differential metabolites in NC, BM, and HM groups. (**F**) Pearson correlation analysis between shared differential metabolites and serum lipid parameters. Different labels (*, **) indicate significant differences (*p* < 0.05, *p*< 0.01).

Combining the metabolites data of serum and microbial, six metabolites were found to be shared in the BM group and significantly different from those in the NC group (Figure 4A). The cluster analysis of these differential metabolites revealed that L-histidine, 3,4-Diaminopyridine, Uric Acid, 4-hydroxybenzaldehyde, 3B-Hydroxy-5-Cholenoic Acid, Erucic Acid, and Proscillaridin had higher expression levels in the NC group compared to those in the BM group, while Muramic Acid and Oleoyl Ethanolamide showed higher expression levels in the BM group than those in the NC group (Figure 4B). Further analysis showed that 4-Hydroxybenzaldehyde significantly positively correlated with LDL-C and TG, while 3B-Hydroxy-5-Cholenoic Acid was significantly positively correlated with LDL-C, TG, and TC. Muramic Acid and Oleoyl Ethanolamide were negatively correlated with LDL-C, TG, TC, and HDL-C, though this correlation was not significant (Figure 4C). Between the HM group and the NC group, 22 metabolites were found to be shared between the serum and the gut microbiome (Figure 4D). Cluster analysis showed that 18 DEMs (6-Methylquinoline, Progesterone, et al.) had higher expression levels in the NC group than those in the HM group, while Seratrodast, Chenodeoxycholic Acid, Docosahexaenoic Acid Ethyl Ester, and Deoxycholic Acid showed higher expression levels in the HM group compared to those in the NC group (Figure 4E). Correlation analysis showed that 6-Methylquinoline was significantly positively correlated with TG; Trans-3-Indoleacrylic acid was significantly positively correlated with LDL-C; Progesterone, Ortho-Hydroxyatorvastatin, and 3B-Hydroxy-5-Cholenoic Acid were significantly positively correlated with LDL-C, TG, and TC; L-Phenylalanine was significantly positively correlated with LDL-C; Aspartame and Dihydrothymine were significantly positively correlated with TG; and Tolycaine, 4-Hydroxybenzaldehyde, and L-Tyrosine were significantly positively correlated with LDL-C and TG. However, Seratrodast, Chenodeoxycholic Acid, Docosahexaenoic Acid Ethyl Ester, and Deoxycholic Acid were negatively correlated with TG, TC, HDL-C, and LDL-C, though these correlations were not significant (Figure 4F). The results suggest that milk intake can increase the levels of Muramic Acid, Oleoyl Ethanolamide, Seratrodast, Chenodeoxycholic Acid, Docosahexaenoic Acid Ethyl Ester, and Deoxycholic Acid in the serum and gut microbiota, which may affect the TG, TC, HDL-C, and LDL-C contents in the serum, and thereby improve the body’s lipid health.

## 4. Discussion

Milk, as an important nutritional source for humans, contains beneficial substances, such as unsaturated fatty acids, amino acids, vitamins, and calcium. However, due to its high fat content, especially saturated fats, the effects of milk intake on blood lipid health remain controversial. Meta-analyses have observed the link between the high-level intake of full-fat milk and the increased risk of coronary heart disease, likely due to its high saturated fat content [21]. In Chinese middle-aged and elderly populations, high-level milk consumption has also found been positively associated with carotid atherosclerosis [22]. However, there are also studies showing that no clear association between milk intake and disease risk [23]. A previous study showed that dairy consumption can prevent fat accumulation in the liver and improve the blood lipid parameters in rats fed a high-fat diet [24]. In a long-term follow-up study of adults for 8 years, higher consumption of total dairy and milk was associated with a lower cardiovascular mortality risk [25]. In a randomized controlled trial, adolescent females who consumed four servings of dairy per day showed a significant increase in lean mass and a reduction in fat mass compared to that of the control group that consumed no dairy [26]. In high-fat diet-induced obese mice, the consumption of Buffalo milk and cow milk reduced their body weight and inhibited liver fat accumulation [27]. Many other epidemiological studies have shown that dairy intake may lead to a lower risk of metabolic syndrome (MetS), type two diabetes, and cardiovascular diseases [28,29,30]. In this study, we found that milk intake can reduce body weight and improve blood lipid health, which is consistent with the point that milk intake, whether Buffalo milk or Holstein cow milk, is beneficial to consumers.

TG, TC, HDL-C, and LDL-C are the critical indicators of blood lipid health, and high values are commonly associated with overweight individuals, which potentially lead to atherosclerotic cardiovascular diseases and insulin resistance [31]. And blood lipids can be altered through dietary interventions [32]. For example, a cohort study on the general population revealed a negative correlation between serum LDL-C levels and milk intake [33]. Following the consumption of various types of dairy products, several metabolic risk markers (e.g., blood pressure, glucose metabolism, lipid profiles, and liver enzymes) were found to be improved [34]. In a cross-sectional population survey, low-fat dairy intake was negatively correlated with LDL-C, although no significant relationship was found with HDL-C or TG, whereas high-fat dairy consumption showed no significant correlation with the lipid parameters [35]. In subjects with mild hypertriglyceridemia, a 15-week dairy intake treatment reduced the TC and TAG levels, while increasing the HDL-C levels [36]. The postprandial TG level measurement is an important predictor of cardiovascular risk [37], and a randomized crossover trial demonstrated a reduction in postprandial TG levels after cheese consumption [38]. In this study, we found that milk intake significantly decreased the levels of TG, TC, and LDL-C in the serum, which is consistent with the above publications, and further confirmed the positive effect of milk intake on blood lipid health.

Diverse studies based on metabolomics have shown that metabolites are associated with metabolic syndrome (MetS)-related diseases such as dyslipidemia [39]. In this study, we identified multiple metabolites that were altered after milk intake, including Oleoyl Ethanolamide (OEA), Chenodeoxycholic Acid (CDCA), Deoxycholic Acid (DCA), and Docosahexaenoic Acid Ethyl Ester (DHA-EE), which are associated with the levels of TG, TC, and LDL-C in the serum. OEA, which is an endogenous unsaturated fatty acid ethanolamide, has been found to exert physiological activity, resulting in reduced body weight and hyperlipidemia in obese rats by activating peroxisome proliferator-activated receptor-α (PPAR-α) [40]. Dietary fat stimulates the production of OEA by small intestinal enterocytes [41]. Research indicates that OEA can decrease the levels of TG and TC in the serum [40]. In this study, we found that the golden hamsters’ intake of Buffalo milk resulted in increased levels of OEA in both the gut microbiome and serum and a negative correlation between OEA and both the serum TG and TC levels, which is consistent with the above reports. In addition, OEA can modulate an increase in the expression level of fatty acid translocase CD36, subsequently regulating feeding behavior and inducing a sense of satiety, thereby having the potential to emerge as a promising therapeutic approach for anti-obesity and hypolipidemic treatments [42]. CDCA is the primary bile acid synthesized in the liver, which facilitates the solubilization and absorption of cholesterol and other lipids in the intestine, thereby maintaining the balance of lipid metabolism [43]. Research has found that CDCA not only participates in the absorption and excretion of lipids, but also functions as a signaling molecule to regulate the process of lipid metabolism [44]. It can activate specific nuclear receptors, such as the Farnesoid X Receptor (FXR), and subsequently modulate the expression of genes related to lipid synthesis and catabolism [45]. This regulatory role aids in maintaining stable lipid levels in the body and preventing the occurrence of abnormal lipid metabolism [46]. In this study, we found a negative correlation between the CDCA and serum lipids, which aligns with this report [47], suggesting that CDCA is a target molecular to affects the serum lipids when consuming milk. DCA is the secondary bile acid produced by intestinal bacteria [48]. A treatment with DCA in human subjects exhibited a trend of reduced cholesterol absorption [49]. Our study showed that milk intake increased the DCA level in both the serum and the gut microbiota, and the DCA level was negatively correlated with the serum biochemical indicators, suggesting that milk intake may improve blood lipid health by improving the DCA level. DHA-EE is an ω-3 polyunsaturated fatty acid ethyl ester that can effectively reduce the level of triglycerides in the plasma [50,51,52]. Medications primarily composed of DHA-EE (such as Omacor, Lovaza, and TAK-085) have been approved for medical indications in many countries, exhibiting good therapeutic effects on people with hypertriglyceridemia and cardiovascular diseases [53,54]. In this study, DHA-EE was also found to improve the serum and the gut microbiota in the milk-treated hamsters, suggesting the positive effect of milk intake on blood lipid health.

The metabolic analysis of the BM and HM groups revealed a common differential metabolite, Miglitol, found in both the serum and the gut microbiome. The expression level of Miglitol was higher in both the serum and the gut microbiome of the HM group compared to those of the BM group. Miglitol is an α-glucosidase inhibitor (α-GIs) and a novel hypoglycemic drug. Other studies have reported that this compound can reduce abdominal fat in patients with metabolic syndrome (MetS) and lower serum LDL-C levels [55]. Animal studies have shown that it plays a role in improving obesity by influencing bile acid (BA) metabolism [56]. In the differential metabolites of the gut microbiome between the BM and HM groups, the expression levels of Niacinamide (NAM) and Linoleic acid (LA) were higher in the HM group compared to those of the BM group. NAM is a precursor to nicotinamide adenine dinucleotide (NAD), and it has been found to prevent hepatic steatosis by inhibiting lipid synthesis [57]. Multi-omics analysis suggests that NAM supplementation can reverse obesity and alleviate obesity-related complications by increasing the NAD levels, enhancing fatty acid oxidation, and boosting glutathione biosynthesis [58]. In obese mice treated with high doses of NAM, significant improvements in hepatic steatosis, inflammation, and glucose tolerance were observed [59]. LA is an essential omega-6 polyunsaturated fatty acid (PUFA) obtained from food [60,61], showing a higher expression level in the HM group than that in in the BM group. The meta-analysis of 2175 participants found that LA can lower the serum levels of TG, TC, and LDL-C [62]. Epidemiological studies have suggested a link between LA supplementation and the reduced risk of cardiovascular diseases and atherosclerosis [63,64,65]. In animal experiments with hypercholesterolemic rats, LA treatment significantly reduced the serum TC, TG, and LDL levels of rats, with effects similar to those of lipid-lowering drugs, indicating LA’s potential in early intervention for hypercholesterolemia [66]. In this study, the TG, TC, and LDL-C levels in the HM group were lower than those in the BM group, while body weight was higher in the HM group, though these changes were not statistically significant. The levels of the three metabolites mentioned above were higher in the serum and gut microbiome of the HM group compared to those of the BM group, which may be related to the lower fat content in Holstein cow milk.

## 5. Conclusions

In this study, we found that milk intake, whether Buffalo milk or Holstein cow milk, can reduce body weight and the serum levels of TG, TC and LDL-C. Further analysis showed that milk intake increased the level of metabolites, such as OEA, CDCA, DCA, and DHA-EE, in both the serum and the gut microbiota. Therefore, milk intake showed a beneficial effect on blood lipid health by altering the metabolites in the serum. This study provides novel evidence that milk consumption is beneficial to health and a reference for guiding people to a healthy diet.

## Data Availability

All datasets used and/or analyzed during the current study are available from the corresponding author upon reasonable request.

## Supplementary Materials

The following supporting information can be downloaded at: https://www.mdpi.com/article/10.3390/metabo14120688/s1, Table S1: Dietary components of experimental diet; Table S2: Analysis of composition in Buffalo milk and Holstein cow milk; Table S3: Body weight of the hamsters; Table S4: Serum TG, TC, LDL-c, and HDL-c levels and LDL-c/HDL-c ratio of the hamsters; Table S5: Serum metabolites; Table S6: Gut microbial metabolites.
